# Examination of gas exchange and blood lactate thresholds in Paralympic athletes during upper-body poling

**DOI:** 10.1371/journal.pone.0205588

**Published:** 2018-10-31

**Authors:** Julia Kathrin Baumgart, Maaike Moes, Knut Skovereng, Gertjan Ettema, Øyvind Sandbakk

**Affiliations:** 1 Centre for Elite Sports Research, Department of Neuroscience and Movement Science, Faculty of Medicine and Health Sciences, Norwegian University of Science and Technology, Trondheim, Norway; 2 Department of Human Movement Sciences, Faculty of Health, Medicine and Life Sciences, Maastricht University, Maastricht, The Netherlands; Nanyang Technological University, SINGAPORE

## Abstract

**Objectives:**

The primary aim was to compare physiological and perceptual outcome parameters identified at common gas exchange and blood lactate (BLa) thresholds in Paralympic athletes while upper-body poling. The secondary aim was to compare the fit of the breakpoint models used to identify thresholds in the gas exchange thresholds data versus continuous linear and curvilinear (no-breakpoint) models.

**Methods:**

Fifteen elite Para ice hockey players performed seven to eight 5-min stages at increasing workload until exhaustion during upper-body poling. Two regression lines were fitted to the oxygen uptake (VO_2_)-carbon dioxide (VCO_2_) and minute ventilation (VE)/VO_2_ data to determine the ventilatory threshold (VT), and to the VCO_2_-VE and VE/VCO_2_ data to determine the respiratory compensation threshold (RCT). The first lactate threshold (LT1) was determined by the first rise in BLa (+0.4mmol·L^-1^ and +1.0mmol·L^-1^) and a breakpoint in the log-log transformed VO_2_-BLa data, and the second lactate threshold (LT2) by a fixed rise in BLa above 4mmol·L^-1^ and by employing the modified D_max_ method. Paired-samples t-tests were used to compare the outcome parameters within and between the different threshold methods. The fit of the two regression lines (breakpoint model) used to identify thresholds in the gas exchange data was compared to that of a single regression line, an exponential and a 3^rd^ order polynomial curve (no-breakpoint models) by Akaike weights.

**Results:**

All outcome parameters identified with the VT (i.e., breakpoints in the VO_2_-VCO_2_ or VE/VO_2_ data) were significantly higher than the ones identified with a fixed rise in BLa (+0.4 or +1.0mmol·L^-1^) at the LT1 (e.g. BLa: 5.1±2.2 or 4.9±1.8 vs 1.9±0.6 or 2.3±0.5mmol·L^-1^,p<0.001), but were not significantly different from the log-log transformed VO_2_-BLa data (4.3±1.6mmol·L^-1^,p>0.06). The outcome parameters identified with breakpoints in the VCO_2_-VE data to determine the RCT (e.g. BLa: 5.5±1.4mmol·L^-1^) were not different from the ones identified with the modified D_max_ method at the LT2 (5.5±1.1mmol·L^-1^) (all p>0.53), but were higher compared to parameters identified with VE/VCO_2_ method (4.9±1.5mmol·L^-1^) and a fixed BLa value of 4mmol·L^-1^ (all p<0.03). Although we were able to determine the VT and RCT via different gas exchange threshold methods with good fit in all 15 participants (mean R^2^>0.931), the continuous no-breakpoint models had the highest probability (>68%) of being the best models for the VO_2_-VCO_2_ and the VCO_2_-VE data.

**Conclusions:**

In Paralympic athletes who exercise in the upper-body poling mode, the outcome parameters identified at the VT and the ones identified with fixed methods at the LT1 showed large differences, demonstrating that these cannot be used interchangeably to estimate the aerobic threshold. In addition, the close location of the VT, RCT and LT2 does not allow us to distinguish the aerobic and anaerobic threshold, indicating the presence of only one threshold in athletes with a disability exercising in an upper-body mode. Furthermore, the better fit of continuous no-breakpoint models indicates no presence of clear breakpoints in the gas exchange data for most participants. This makes us question if breakpoints in the gas exchange data really exist in an upper-body exercise mode in athletes with disabilities.

## Introduction

In able-bodied endurance athletes performing lower-body or whole-body exercise, gas exchange and blood lactate (BLa) threshold concepts are well-established in the diagnosis of endurance performance as well as in the prescription of systematic training with different exercise intensity zones [1]. Two thresholds are commonly described in the literature: 1) The aerobic threshold (AT)–determined by the ventilatory threshold (VT) or the first lactate threshold (LT1)–separates low- from moderate-intensity exercise [2, 3]. 2) The anaerobic threshold (ANT)–determined by the respiratory compensation threshold (RCT) or the second lactate threshold (LT2)–separates moderate- from high-intensity exercise [2, 3]. However, to what extent the outcome parameters identified at the VT and LT1 as well as the RCT and LT2 coincide in Paralympic sitting sport athletes who exercise in an upper-body mode remains to be investigated.

Various methods have been employed to determine the VT and the RCT, as well as the LT1 and the LT2 [3–6]. The VT is based on a disproportionate increase (i.e. a breakpoint) in carbon dioxide production (VCO_2_) and minute ventilation (VE) in relation to oxygen uptake (VO_2_) [3, 7], and the LT1 on an onset in BLa concentration above resting levels that marks the beginning of exercise [5] or on a breakpoint in the log-log transformed VO_2_-BLa data [4]. Even though these physiological changes occur above the VT and LT1, the body is still able to maintain equilibrium at intensities up to the ANT, and aerobic metabolism (indicated by measurements of oxygen uptake and the corresponding energy equivalent) reflects overall energy expenditure [2]. The ANT marks the point beyond which any attempt of the body to maintain metabolic equilibrium at a constant rate of work fails [6]. The RCT is based on a disproportionate increase (i.e. a breakpoint) of VE in relation to VCO_2_ [3], a mechanism that has been suggested to correspond with the point where BLa starts to accumulate with constant workload [6]. In contrast, it has been argued that the changes in gas exchange with increasing work rate are continuous transitions where fatigue gradually accumulates rather than clear breakpoints [8].

The assumption that the VT corresponds with the LT1, and the RCT with the LT2, are based on the initial studies by Beaver et al. [3] and Wassermann et al. [6, 9, 10] from the 1980’s. However, there has been a continuous debate around the existence of and the physiological link between these different thresholds [2, 11–14]. Although physiological parameters identified at the VT and LT1, and at the RCT and LT2 have shown high correlations in able-bodied participants during cycling and running in some studies [15, 16], others find low correlations [17]. In wheelchair basketball and wheelchair rugby athletes with a spinal cord injury, the % of VO_2peak_ was lower at the LT1 compared to the VT, whereas it did not significantly differ at the LT2 and RCT [18]. In contrast, in able-bodied swimmers, there were no significant differences in physiological outcome parameters at the LT1 and the VT [19].

Whereas a range of studies have investigated the VT during upper-body exercise in able-bodied participants and participants with a disability [20–29], knowledge is limited on whether gas exchange and BLa threshold concepts can be used interchangeably in athletes with disabilities who exercise in an upper-body mode, or whether breakpoints exist in the gas exchange data of these athletes. Therefore, the primary aim of this study was to compare physiological and perceptual outcome parameters at the gas exchange and BLa thresholds in the data obtained from Paralympic athletes while upper-body poling. The secondary aim was to compare the fit of breakpoint models used to identify gas exchange thresholds with continuous linear or curvilinear (no-breakpoint) models.

## Methods

### Participants

Fourteen male and one female endurance-trained Norwegian Para ice hockey players participated in this study. Anthropometrics and training hours per month of the participants are depicted in Table 1. All participants were healthy and free of injuries at the time of testing. The study was approved by the Norwegian Data Protection Authority and conducted in accordance with the Declaration of Helsinki. All participants signed an informed consent form prior to voluntarily take part in the study, and were made aware that they could withdraw from the study at any point without providing an explanation.

**Table 1:** Sex, age, anthropometric and disability characteristics as well as monthly training hours of the 15 Norwegian national team Para ice hockey players participating in this study.

	Sex	Age (years)	Body mass (kg)	Height (cm)	Disability (level of injury)	Training hrs/month
1	Male	53	83.3	186	Paraplegia (Th12-L1)	25
2	Male	18	75.7	160	Spina bifida (L5)	49
3	Male	27	61.0	160	Athrogryposis multiplex congenita	63
4	Male	31	69.4	184	Hereditary spastic paraplegia	45
5	Male	28	90.0	173	Paraplegia (Th10)	26
6	Male	21	70.4	164	Spina bifida (ns)	59
7	Male	33	70.5	160	Spina bifida (Th12)	67
8	Male	34	75.3	173	Paraplegia (Th11-12)	48
9	Female	22	70.0	167	Spina bifida (L3-S1)	33
10*	Male	22	63.4	164	Paraplegia (Th11-12)	28
11*	Male	18	64.2	154	Spina bifida (ns)	54
12	Male	20	68.0	186	Paraplegia (Th12)	40
13	Male	20	77.0	163	Cerebral Palsy (motor only)	23
14	Male	28	66.5	173	Amputation (single leg above the knee)	80
15	Male	32	63.2	165	Paraplegia (ns)	56
Mean ± SD		27.1±8.9	71.2±8.0	170±10	-	47±18

* Players are from the Norwegian national B-team

All other players are from the Norwegian national A-team.

Thoracic (Th), lumbar (L), sacral (S), not specified (ns)

### Experimental design

The testing consisted of two consecutive test days at similar test times, during which participants performed an incremental test to exhaustion on day one, followed by seven to eight 5-min stages at gradually increasing effort for each stage until exhaustion on day two. All tests were performed in upper-body poling on a Concept2 ski ergometer 1 (Concept2, Inc., Morrisville, USA, http://www.concept2.com/service/skierg/skierg-1), while sitting in an ice sledge hockey seat.

### Test set-up

**Fig 1:**
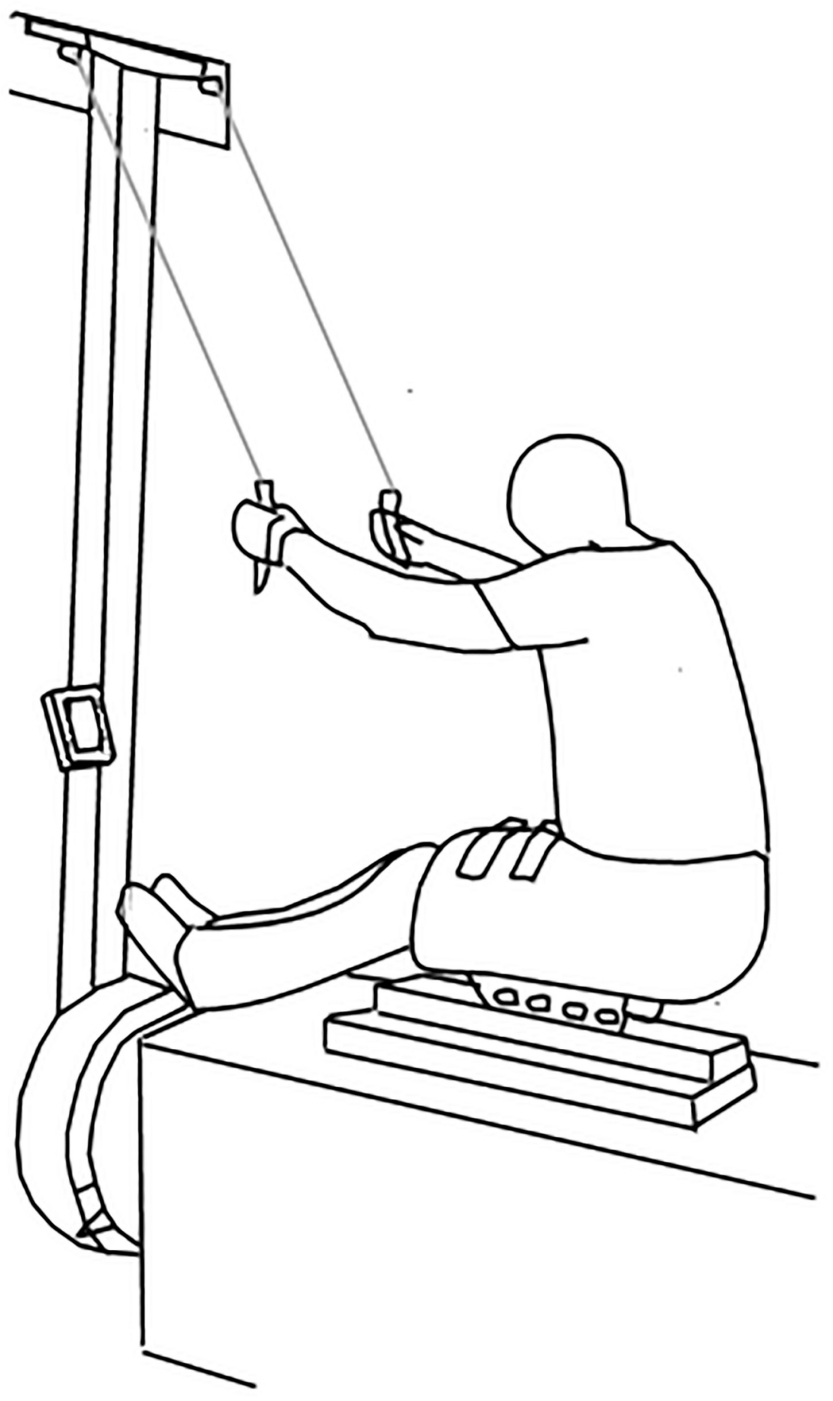
Test set-up. The participants were strapped in around the hips and thighs in an ice sledge hockey seat mounted on a platform in front of the Concept2 ski-ergometer.

After being equipped with an oro-nasal mask (Hans Rudolph Inc, Kansas City, MO, USA) and a heart rate monitor (Polar Electro Inc., Port Washington, NY, USA), the participants were tightly strapped around the thighs and hips into an ice sledge hockey seat that was mounted on a wooden platform (Fig 1). The distance of the seat to the Concept2 ski ergometer and the position of the feet depended on personal preference but was the same for test day one and two. The ski ergometer uses wind resistance, which is generated by the spinning flywheel. The ski ergometer has a spiral damper with settings from one to ten, which works like a gearing system. We had this damper set at “eight” for all participants. Power output was measured with the ergometer’s software, which was previously validated with force and velocity measurements using a force cell (Noraxon USA inc., Scottsdal, AZ, USA) and the Oqus cameras of the Qualisys motion capture system (Qualisys AB, Gothenburg, Sweden) as described by Hegge et al. [30]. The Metamax II ergospirometer CORTEX Biophysik GmbH, Leipzig, Germany) was calibrated against a known mixture of gases (16% O_2_ and 4% CO_2_) and ambient air prior to the testing procedure of every second participant. Before each athlete was tested, the flow transducer was calibrated with a 3 L syringe and then connected to the oro-nasal mask, which allowed for the measurement of breath-by-breath respiratory parameters.

### Test protocol

The participants were instructed to refrain from heavy training and alcohol consumption 24 hours before, caffeine intake the day of, and food intake two hours before testing. Additionally, the participants were instructed to void their bladder directly before arriving at the laboratory. A questionnaire was filled out on each of the two test days to monitor if the participants followed these instructions, as well as to exclude any prior illness or injury that might have interfered with the testing.

#### Test day one

A standardized warm-up of five 5-min submaximal stages with a 2- to 3-min break between stages was performed in the upper-body poling mode at an overall rating of perceived exertion (RPE) of 7 (very light), 9 (very light), 11 (light), 13 (somewhat hard) and 15 (hard). Next to serving as a warm-up, the submaximal stages were used to familiarize the participants with the use of the Borg scale [31] to indicate RPE after the incremental test and each of the 5-min stages on day two. After a 5-min break, the incremental test started at the individual power output of the third submaximal stage (rounded to the nearest 10-point value), and participants were instructed to continuously increase power output by 10 W every 30 s. The test was terminated when the participant, despite strong verbal encouragement, could no longer maintain the required power output of the 30-s stage and the VO_2_ values either plateaued or decreased (a drop of more than 2 mL·kg^-1^·min^-1^). After the incremental test, participants recovered passively for five min and actively for three min (at the power output of the first submaximal stage). They then performed a verification stage at a 10% higher power output than the peak power output of the incremental test (rounded to the nearest 10-point value) to verify the attainment of a true VO_2peak_ [32]. The verification stage was terminated when the participant dropped more than 10% of target power output for more than five s.

#### Test day 2

Seven to eight 5-min stages were performed with a 2- to 3-min break between stages and in the same upper-body poling mode. The first stage started at 20% of the individual peak power output obtained during the incremental test on day one, with increases of 10% (of the individual peak power output) for each consecutive stage. The last stage was terminated when the participant, despite strong verbal encouragement, could no longer maintain the power output of that stage and dropped more than 10% in the target power output for longer than five s. The intermittent exercise protocol was chosen to take a BLa sample from the fingertip in between stages. The duration of five min per stage was chosen, since in an upper-body mode two to three min are needed to achieve steady-state of physiological outcome parameters [33].

### Outcome measurements

Heart rate was measured every second with a Polar heart rate monitor, and respiratory parameters (i.e., VO_2_, VCO_2_, VE, and respiratory exchange ratio (RER)) were measured breath-by-breath and averaged over 10 s by the in-built software of a Metamax II. A blood sample was taken from the fingertip and BLa analysed with a Lactate Pro device (Arkray Inc., Japan) at rest and directly after each of the submaximal stages on day one and day two, and one and three min after the incremental test and the verification stage on day one as well as the last stage of day two. Overall RPE was recorded after each of the submaximal stages on day one and two, as well as after the incremental test on day one and the last stage on day two. Power output was displayed per stroke and saved as 20-s averages during the submaximal stages on day one and day two by the in-built Concept2 software (Concept2, Morrisville, VT, USA). Peak power output during the incremental test and during the verification stage was registered as the highest 30-s average.

### Data analysis

#### Data processing

Peak power output and gas exchange outcome parameters were calculated as the highest 30-s moving average and peak heart rate (HR_peak_) as the highest 3-s moving average of the incremental test performed on test day one. The gas exchange, heart rate and power output data of the last two min (12 x 10-s averages) of each complete 5-min stage conducted on test day two was included for data analysis in MATLAB (R2016a; Mathworks Inc., Natick, MA). The analyses in the following were based on the concatenated 2-min gas exchange data for the VT and RCT and on the BLa values after each 5-min stages for the LT1 and LT2.

Different methods were used to determine both the VT and the RCT, as well as the LT1 and the LT2. For the determination of the VT, VO_2_ was plotted against VCO_2_ (V-slope method) [3] as well as time against VE/VO_2_ and VE/VCO_2_ (ventilatory equivalent method) [7] and two regression lines fit to the data. For a valid detection of the VT with the ventilatory equivalent method, the VE/VO_2_ had to increase before an increase in VE/VCO_2_ [15, 34]. For the detection of the RCT, VCO_2_ was plotted against VE [3] and two regression lines fit to the data. The LT1 was determined in two different ways: the first fixed rise in BLa concentration by 0.4 and 1 mmol·L^-1^ above the lowest individual BLa value [5, 35]. Additionally, the LT1 was determined by breakpoints in the log-log transformed VO_2_-BLa relationship [4]. The LT2 was determined by a fixed BLa concentration of 4 mmol·L^-1^ [36]. Additionally, the LT2 was determined by the modified D_max_ method, which identifies the point on the 3^rd^ order polynomial curve fitted to the BLa values that yields the maximal perpendicular distance to the straight line formed by the first stage with an increase of 0.4 mmol·L^-1^ and the BLa measured after the last stage [5]. Outcome parameters (% of peak power output, % of VO_2peak_, % of HR_peak_, as well as BLa and RPE) were interpolated at the thresholds identified with each of the above described methods used to determine the VT, LT1, RCT and LT2.

#### Statistical analyses

Paired-samples t-tests were used to compare the physiological and perceptual outcome parameters within the VT, LT1, RCT and LT2, and between all four different thresholds. Pearson’s r was used to investigate relationships between the outcome parameters identified with the different methods used to determine VT, LT1, RCT and LT2. Ranges of 0.26–0.49, 0.50–0.69, 0.70–0.89 and 0.90–1.0 were used to indicate low, moderate, high and very high correlations according to Munro’s criteria [37]. An α level of 0.05 was used to indicate statistical significance.

To compare the fit of breakpoint models versus continuous linear or curvilinear (no-breakpoint) models to the gas exchange data, two regression lines (Eq 1) versus a single linear regression line (Eq 2), an exponential curve (Eq 3), and a 3^rd^ order polynomial curve (Eq 4) were fitted to the VO_2_-VCO_2_, VE/VO_2_, VE/VCO_2_, and VCO_2_-VE data by linear least squares fitting.

$$y=\{\begin{array}{c} a_{1}+b_{1}\;x,\;t<k\\ a_{2}+b_{2}\;x,\;t\geq k \end{array}$$ (1)

y=a+bx (2)

$$y=a+c\cdot exp\left(\frac{x+g}{d}\right)$$ (3)

$$y=a+b_{1}\;x+b_{2}\;x^{2}+b_{3}\;x^{3}$$ (4)

*y* is the variable of interest, *a* the y-axis offset, *b* the slope coefficients, *c* and *d* spreading coefficients, *g* the x-axis offset and *k* the point where the first and the second regression line of the piecewise function cross. To compare the fit of the four models, the Akaike information criterion (AIC) (Eq 5) [38] and the Akaike weights (*w*_*i*_) (Eq 7) for each model *i* relative to the set of *R* candidate models were calculated based on the delta AIC (Δ_i_) (Eq 6) [39, 40].

$$AIC=n∙log\left(\frac{SS_{er}}{n}\right)+2∙K$$ (5)

$$Delta\;AIC=\Delta_{i}=AIC_{i}-AIC_{2reg}$$ (6)

$$AIC\;weight=w_{i}=\frac{{exp}^{\left(-\frac{\Delta i}{2}\right)}}{\sum_{r=1}^{R}{exp}^{\left(-\frac{\Delta r}{2}\right)}}$$ (7)

*n* is the number of data points, *SS*_*er*_ the error sums of squares, and *K* the number of parameters +1 of each model. Our rationale was that a better fit of the two regression lines (breakpoint model) as compared to the linear/curvilinear models (continuous no-breakpoint models), would suggest the presence of a breakpoint.

## Results

**Fig 2:**
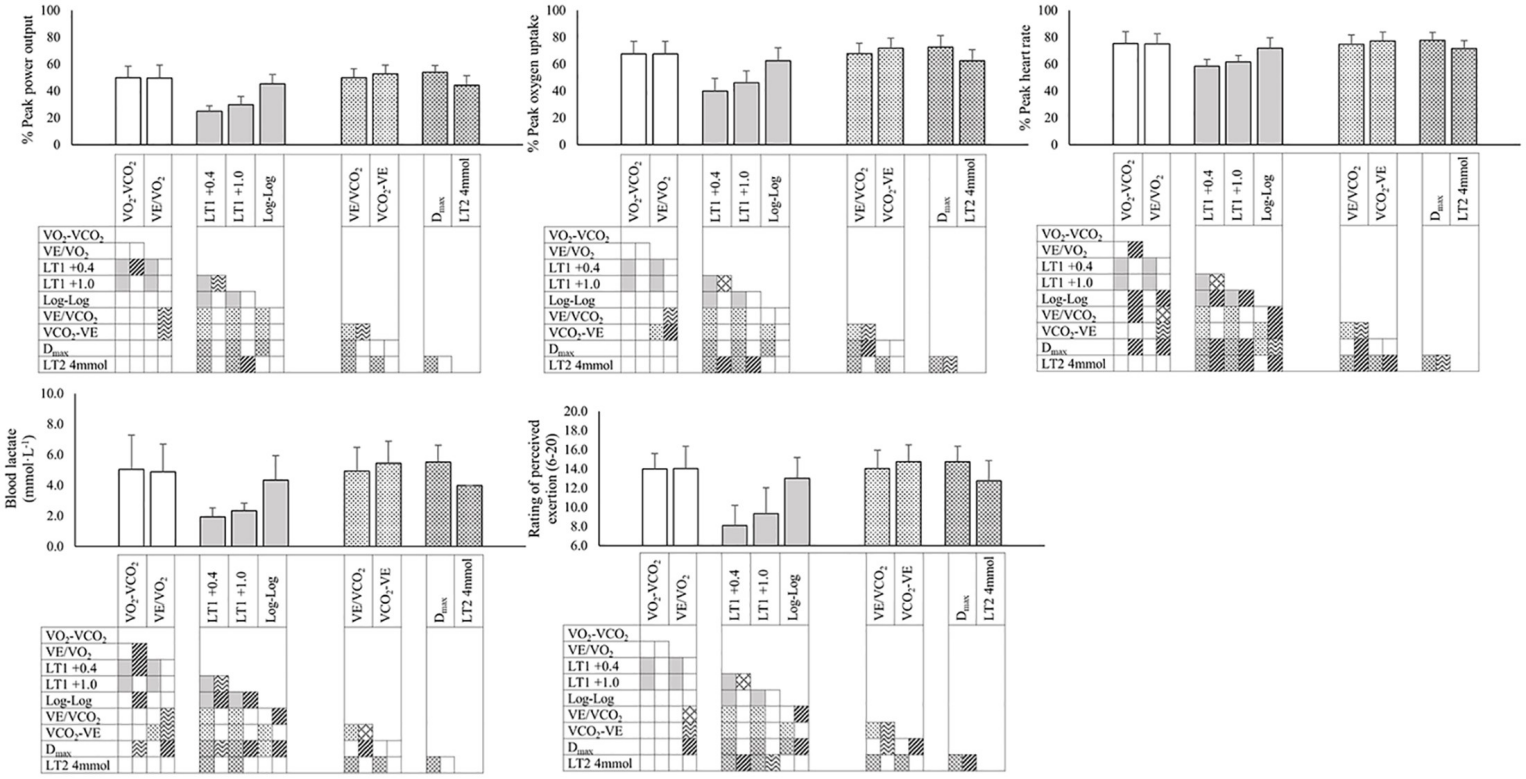
Outcome parameters at the VT, LT1, RCT and LT2. Outcome parameters at the aerobic threshold are determined by the V-slope (VO_2_-VCO_2_ data) and the ventilatory equivalent method (VE/VO_2_ data) to identify the VT, and a fixed rise in BLa of 0.4 and 1.0 mmol·L^-1^ and the log-log transformed VO_2_-BLa data to identify the LT1. Outcome parameters at the anaerobic threshold are determined by the ventilatory equivalent method (VE/VCO_2_ data) and the respiratory compensation point (VCO_2_-VE data) to identify the RCT, and the modified D_max_ method and a fixed BLa value of 4 mmol·L^-1^ to identify the LT2. The data used is from 15 elite Para ice hockey players following a protocol with stepwise increases in workload every 5 min while upper-body poling. *The data presented is the mean of all 15 participants and error bars denote +1 SD*. *Filled squares in the left columns denote significant differences between methods at an alpha level of 0*.*05*. *Filled squares in the right columns denote significant correlations of methods (upward diagonal = moderate*, *zig zag = high*, *diamond outline = very high) at an alpha level of 0*.*05*.

The outcome parameters identified with different methods used to determine the VT, LT1, RCT and LT2 (Fig 2) are presented as percentage of the respective peak power output, VO_2peak_ and peak HR obtained during the incremental test (Table 2).

**Table 2:** Mean ± SD (95% CI) peak power output and peak physiological and perceptual outcome parameters.

	Peak values
**Peak power output** (W)	144 ± 37 (125–163)
**VO**_**2peak**_ (mL·kg^-1^·min^-1^)	36 ± 7 (32–39)
**HR**_**peak**_ (beats·min^-1^)	188 ± 12 (182–194)
**Blood lactate** (mmol·L^-1^)	14.4 ± 1.5 (13.7–15.2)
**RPE** (6–20)	19.7 ± 0.5 (19.4–19.9)

The data was collected during an incremental test to exhaustion while upper-body poling of 15 Norwegian Para ice hockey players.

Peak oxygen uptake (VO_2peak_), peak heart rate (HR_peak_), rating of perceived exertion (RPE)

All outcome parameters identified at VT with either the V-slope or the ventilatory equivalent method were significantly higher than the ones at both the LT1 (+0.4) and LT1 (+1.0) (all p<0.001), but not significantly different from the ones identified with the log-log transformed VO_2_-BLa method (all p>0.06) (Fig 2). Additionally, most of the outcome parameters identified at the VT did not significantly correlate with the corresponding ones at LT1 (+0.4) or LT1 (+1.0) (exception: power output and BLa at LT1 (+0.4): r>0.55, p<0.04; all other outcome parameters: r<0.38, p>0.16) (S1 File, sheet “correlations”). All outcome parameters at LT1 (+0.4) and LT1 (+1.0) were highly or very highly correlated (all r>0.83, p<0.001). In addition, some of the outcome parameters identified with breakpoints in the log-log transformed VO_2_-BLa moderately correlated with the outcome parameters identified by the V-slope method (HR: r = 0.64, p = 0.01; BLa: r = 0.54, p = 0.04) and the breakpoints in the VE/VO_2_ data of the ventilatory equivalent method (HR: r = 0.54, p = 0.04).

The outcome parameters identified with breakpoints in the VCO_2_-VE data at the RCT (e.g. BLa: 5.5±1.4 mmol·L^-1^) were not significantly different from the ones identified with the modified D_max_ method at the LT2 (5.5±1.1 mmol·L^-1^) (all p>0.53), but were higher compared to parameters identified with VE/VCO_2_ method (4.9±1.5 mmol·L^-1^) and a fixed BLa value of 4 mmol·L^-1^ (all p<0.03). Furthermore, there was no significant difference between the outcome parameters identified with V-slope method used to determine the VT and the ones identified with breakpoints in the VE/VCO_2_ and VCO_2_-VE data used to determine the RCT (p>0.22). However, most outcome parameters identified at the breakpoints in the VE/VO_2_ and VE/VCO_2_ data (ventilatory equivalent method) were highly or very highly correlated with those identified at the breakpoints in the VCO_2_-VE data (RCT) (exception: % of VO_2peak_ r = 0.67, p = 0.006; all other outcome parameters: r>0.73, p<0.01) (Fig 2). In addition, most outcome parameters identified at the thresholds in the VE/VCO_2_ data were moderately to highly correlated with the same outcome parameters at the thresholds identified with the modified D_max_ method (exception: % of peak power output: r = 0.43, p = 0.11; all other outcome parameters: r>0.57, p<0.03). Furthermore, there was no significant difference between the outcome parameters identified with the log-log transformed VO_2_-BLa method used to determine the LT1 and at a fixed BLa concentration of 4 mmol·L^-1^ used to determine the LT2 and (all p>0.43).

**Fig 3:**
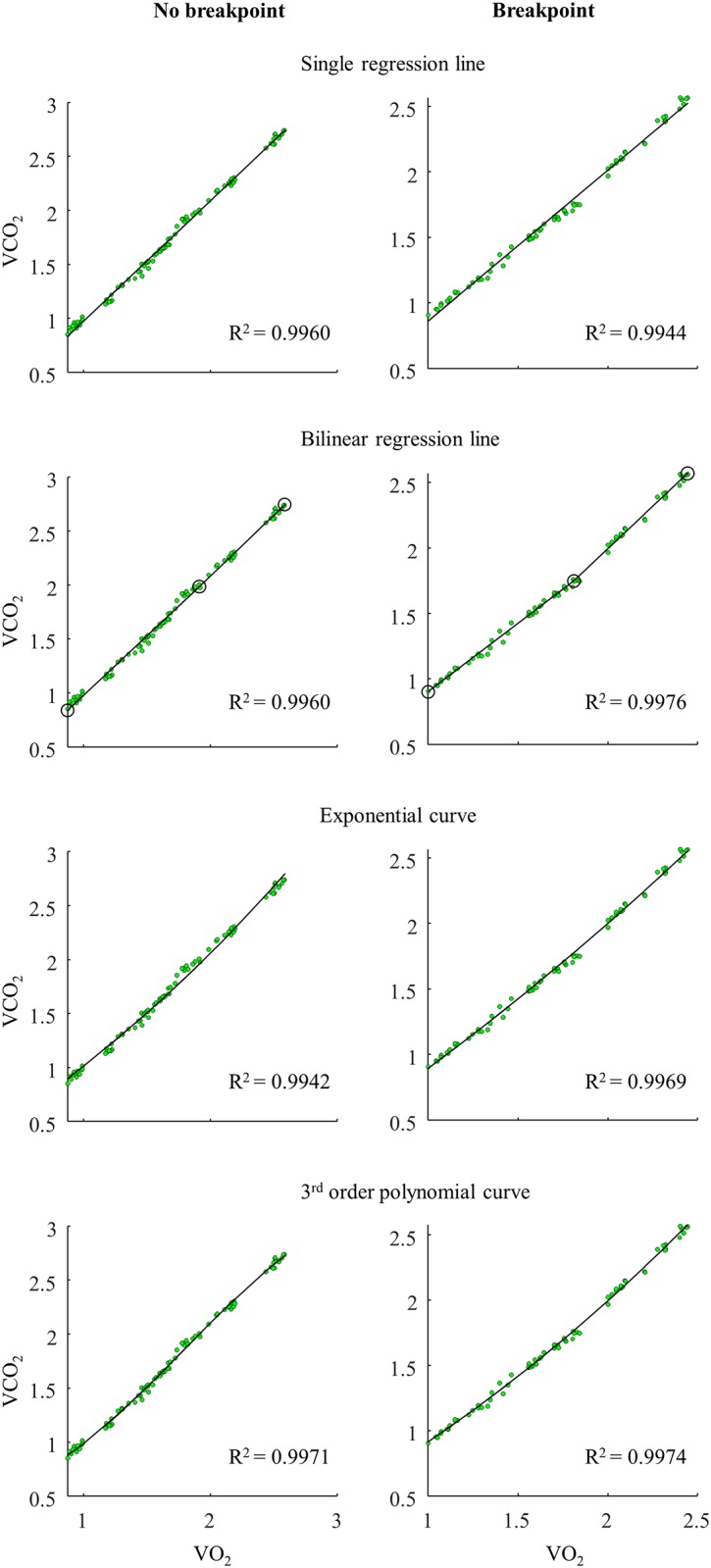
Exemplary VO_2_-VCO_2_ plots. The VO_2_-VCO_2_ data was fitted with a single regression line, a bilinear regression line, an exponential curve, and a 3^rd^ order polynomial curve for an athlete without breakpoint (the four plots to the left) and with suggested breakpoint presence (the four plots to the right). (Note that the plots of the five athletes with a suggested breakpoint also show a rather linear increase in the VO_2_-VCO_2_ relationship). *Oxygen uptake (VO*_*2*_*)*, *carbon dioxide production (VCO*_*2*_*)*.

**Fig 4:**
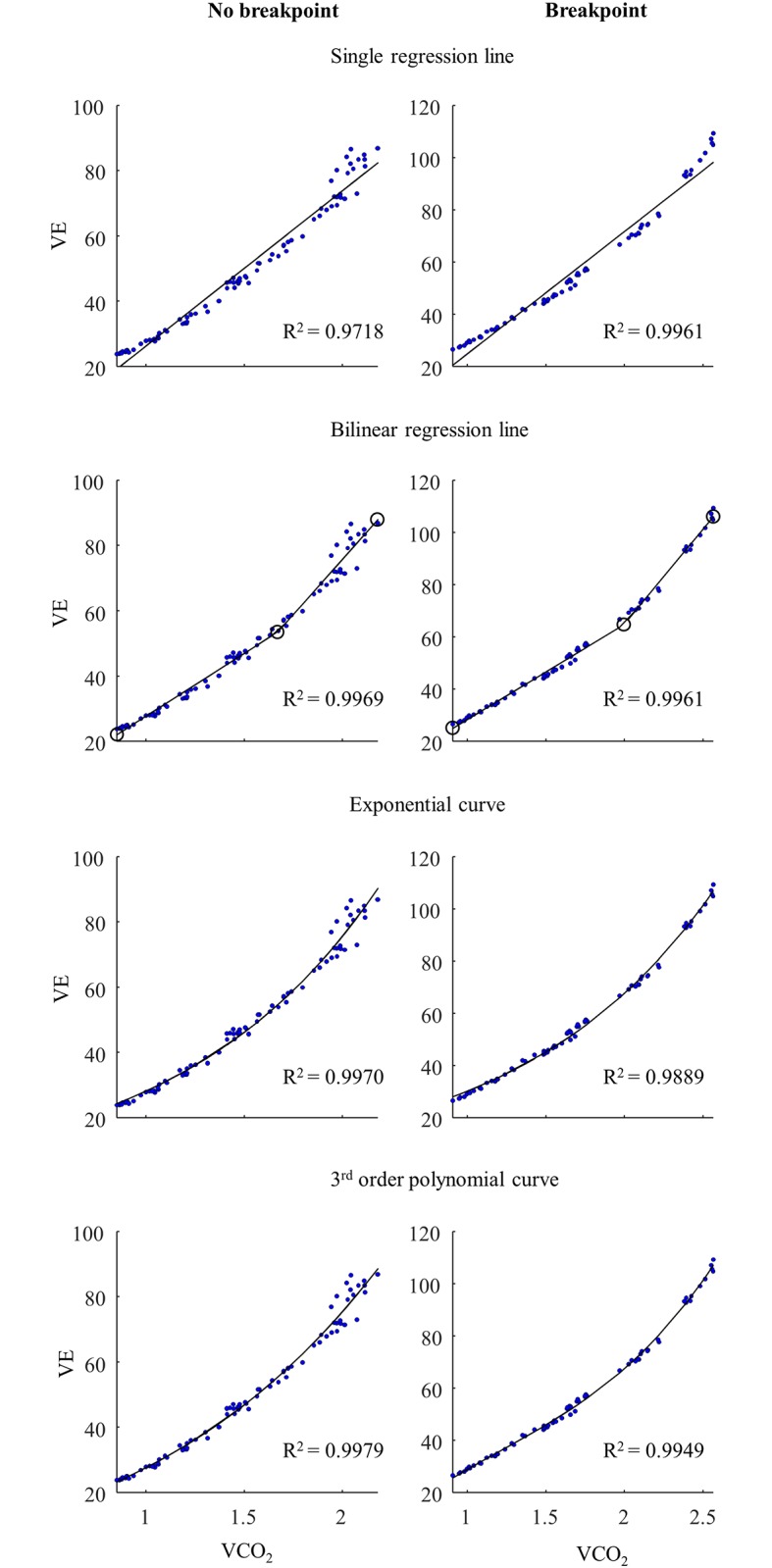
Exemplary VCO_2_-VE plots. The VCO_2_-VE data was fitted with a single regression line, a bilinear regression line, an exponential curve, and a third order polynomial curve for an athlete without breakpoint (the four plots to the left) and with suggested breakpoint presence (the four plots to the right). (Note that the plots of the five athletes with a suggested breakpoint show a rather curvilinear increase in the VCO_2_-VE relationship). *Carbon dioxide production (VCO*_*2*_*)*, *minute ventilation (VE)*.

For the gas exchange data, all fitting procedures for the VO_2_-VCO_2_ and the VCO_2_-VE plots, including the single linear regression line, showed very good fit on the data for all 15 participants (mean r^2^>0.97) (Table 3). However, the fit of the breakpoint model compared to the continuous no-breakpoint models on the VO_2_-VCO_2_ and the VCO_2_-VE data was only better among five participants. Accordingly, the continuous no-breakpoint models had 71% and 68% probability of being the best models for the VO_2_-VCO_2_ and the VCO_2_-VE data, respectively (Table 4). Exemplary VO_2_-VCO_2_ and VCO_2_-VE plots are illustrated in Figs 3 and 4, respectively.

**Table 3:** The coefficient of determination (mean r^2^ ± SD (range) for the two regression lines (breakpoint model) and the single regression line, exponential and 3^rd^ order polynomial curve (continuous no-breakpoint models) fitted to the gas exchange data of 15 elite Para ice hockey players following a protocol with stepwise increases in workload every 5 min while upper-body poling.

	Two regression lines	Single regression line	Exponential curve	3^rd^ order polynomial curve
**VO**_**2**_**-VCO**_**2**_ **plots**	0.995 ± 0.005 (0.993–0.998)	0.994 ± 0.005 (0.991–0.996)	0.993 ± 0.005 (0.991–0.996)	0.996 ± 0.005 (0.993–0.998)
**VE/VO**_**2**_ **plots**	0.931 ± 0.069 (0.896–0.966)	0.764 ± 0.094 (0.716–0.811)	0.919 ± 0.064 (0.886–0.951)	0.932 ± 0.064 (0.900–0.964)
**VE/VCO**_**2**_ **plots**	0.940 ± 0.044 (0.918–0.962)	0.700 ± 0.142 (0.628–0.772)	0.920 ± 0.044 (0.898–0.942)	0.940 ± 0.041 (0.919–0.961)
**VCO**_**2**_**-VE plots**	0.995 ± 0.003 (0.994–0.997)	0.968 ± 0.015 (0.960–0.976)	0.992 ± 0.006 (0.989–0.994)	0.996 ± 0.003 (0.994–0.998)

Oxygen uptake (VO_2_), carbon dioxide production (VCO_2_), minute ventilation (VE)

**Table 4:** Akaike weights (*w*_*i*_) representing a measure of strength of evidence for probability of best fit of the two regression lines (breakpoint model) and the single regression line, exponential and 3^rd^ order polynomial curve (continuous no-breakpoint models) (mean *w*_*i*_ ± SD (95% CI) [# of participants with better fit of the respective model compared to the two regression lines]) fitted to the gas exchange data of 15 elite Para ice hockey players following a protocol with stepwise increases in workload every 5 min while upper-body poling.

	Two regression lines	Single regression line	Exponential curve	3^rd^ order polynomial curve
**VO**_**2**_**-VCO**_**2**_ **plots**	0.29 ± 0.35 (0.11–0.46)	0.07 ± 0.18 (-0.03–0.16) [#0]	0.03 ± 0.10 (-0.01–0.08) [#0]	0.61 ± 0.37 (0.42–0.79) [#10]
**VE/VO**_**2**_ **plots**	0.41 ± 0.45 (0.18–0.64)	0.00 ± 0.00 (0.00–0.00) [#0]	0.13 ± 0.24 (0.01–0.26) [#2]	0.46 ± 0.40 (0.25–0.66) [#7]
**VE/VCO**_**2**_ **plots**	0.47 ± 0.49 (0.22–0.72)	0.00 ± 0.00 (0.00–0.00) [#0]	0.09 ± 0.19 (-0.01–0.19) [#2]	0.44 ± 0.43 (0.22–0.66) [#6]
**VCO**_**2**_**-VE plots**	0.31 ± 0.44 (0.09–0.54)	0.00 ± 0.00 (0.00–0.00) [#0]	0.00 ± 0.01 (0.00–0.01) [#0]	0.68 ± 0.44 (0.46–0.91) [#10]

Oxygen uptake (VO_2_), carbon dioxide production (VCO_2_), minute ventilation (VE)

**Fig 5:**
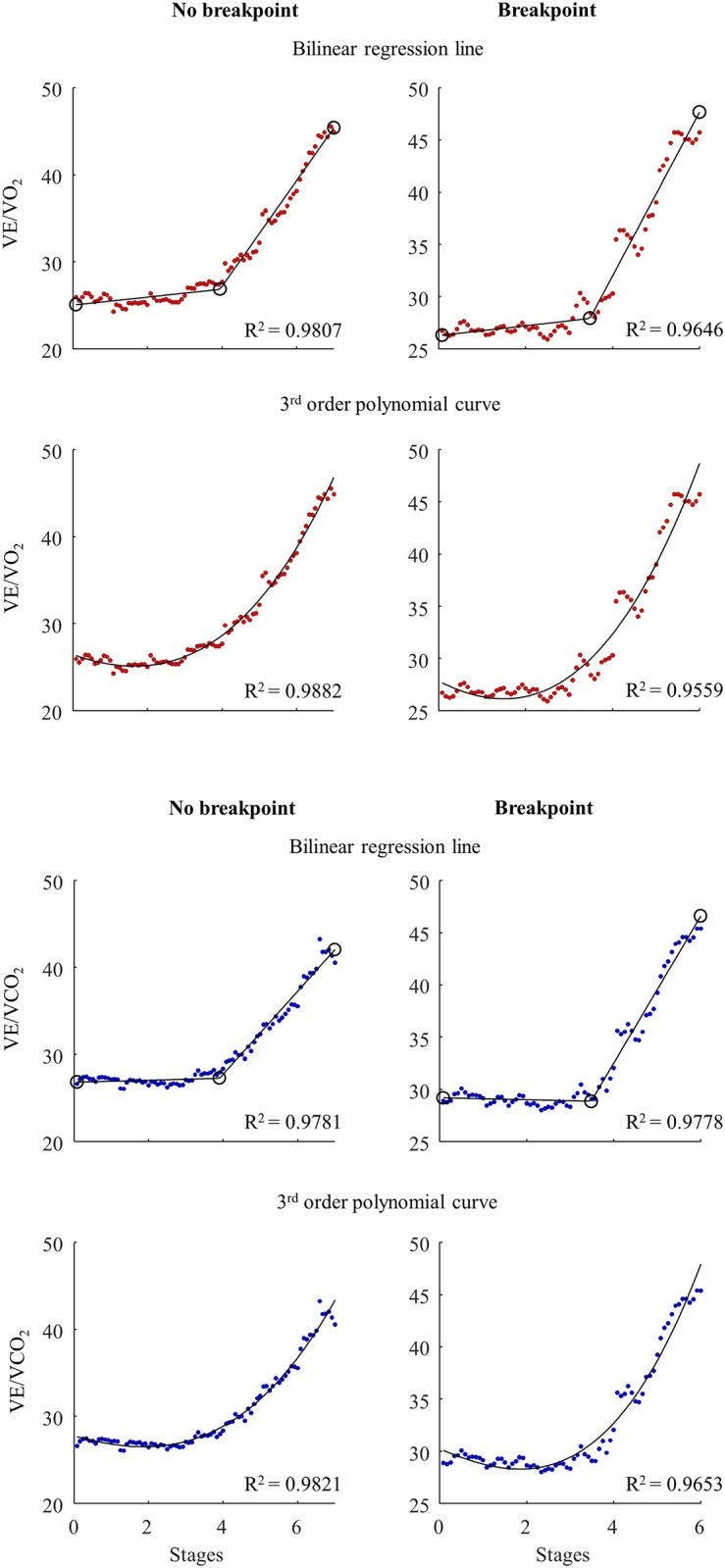
Exemplary VE/VO_2_ and VE/VCO_2_ plots. Exemplary VE/VO_2_ data fitted with a bilinear regression line and a 3^rd^ order polynomial curve for an athlete without breakpoint (upper two plots to the left) and with suggested breakpoint presence (upper two plots to the right). Exemplary VE/VCO_2_ data fitted with a bilinear regression line and a 3^rd^ order polynomial curve for an athlete without breakpoint (lower two plots to the left) and with suggested breakpoint presence (upper two plots to the right). *Oxygen uptake (VO*_*2*_*)*, *carbon dioxide production (VCO*_*2*_*)*, *minute ventilation (VE)*.

In the gas exchange data displayed in the VE/VO_2_ plots and the VE/VCO_2_ plots, the breakpoint model fitted better than the continuous no-breakpoint models in six and seven of the athletes, respectively (Fig 5). Accordingly, it is unclear if in general the breakpoint (41 and 47%, respectively) or continuous no-breakpoint (59 and 53%, respectively) models fit the VE/VO_2_ and the VE/VCO_2_ data best (Table 4). The rise in VE/VO_2_ occurred earlier than the VE/VCO_2_ only in four athletes (S1 Fig). The VT detection by the VE/VO_2_ relationship was, therefore, only valid in these four athletes. In none of these four athletes, did the breakpoint model fit the VE/VO_2_ data better than the continuous no-breakpoint models.

## Discussion

The main aim of this study was to compare physiological and perceptual outcome parameters identified with common gas exchange and BLa thresholds methods used to determine the VT, LT1, RCT and LT2 in Paralympic athletes while upper-body poling. Furthermore, we compared the fit of breakpoint models used to determine gas exchange thresholds to the fit of continuous linear or curvilinear (i.e., no-breakpoint) models. The LT1 occurred at much lower exercise intensity than the VT although both are used as indicators of AT, whereas there were no or minor differences between the methods used to identify the RCT and LT2 that determine the ANT. Furthermore, the RCT and LT2 did not differ from the VT. In addition, the outcome parameters corresponding to the LT1 and LT2 using the log-log transformed VO_2_-BLa data and the modified D_max_ method, respectively, were significantly higher than ones identified with fixed BLa values at the LT1 and LT2 (i.e., rise in BLa of +0.4/1.0 at LT1 or BLa concentration of 4 mmol·L^-1^ at LT2). We were able to determine breakpoints at the VT and RCT with different gas exchange methods with good fit in all 15 participants, although continuous no-breakpoint models showed even better fit for the majority of participants.

The physiological and perceptual outcome parameters identified with a fixed rise in BLa at the LT1 were significantly lower than the ones at the VT, and the outcome parameters using these methods only low or moderately correlated with each other. Overall, this indicates that these two thresholds cannot be used interchangeably to determine the AT. In addition, thresholds identified by a fixed BLa increase at the LT1 were significantly lower compared with the breakpoints identified in the log-log transformed VO_2_-BLa data, showing that individually adjustable BLa methods did not correspond with fixed methods in determining the LT1. The early occurrence of a rise in BLa in upper-body exercise is in accordance with Beneke et al. [41], who found BLa to be higher at a given workload in activities involving smaller muscle mass, where power output per kg of active muscle mass and, thus, local metabolic stress is increased compared to lower body exercise. In addition, BLa accumulation after cessation of exercise was shown to be faster in individuals with a spinal cord injury as compared to able-bodied individuals [42]. However, although outcome parameters identified with breakpoints in the log-log transformed VO_2_-BLa data are not significantly lower than the ones identified at the VT, outcome parameters identified with methods using fixed BLa values to identify the LT1 are much lower than the VT.

As estimates of the ANT, the outcome parameters identified with the D_max_ method to determine LT2 did not significantly differ from the ones identified with breakpoints in the VCO_2_-VE data at the RCT, whereas most of the outcome parameters identified with breakpoints in the VE/VCO_2_ data were significantly lower than these. However, the outcome parameters identified by the latter method differ only marginally from the two other ANT methods (VCO_2_-VE, D_max_), indicating that the exercise intensity where a disproportionate increase in BLa and in VE occurs is relatively similar. Note that we decided to not correct for multiple comparisons and rather present the uncorrected p-values from paired samples t-tests instead. Although we are aware of the subsequent increased chances of making a type 1 errors, the decreased chances of making a type a type 2 errors were regarded more important, which is in accordance with Rothman [43]. However, if Bonferroni corrections would have been used in this specific case, there would have been no significant differences between the outcome measures identified at with these three methods.

Furthermore, most of the outcome parameters identified with the different methods at the LT2 and RCT are low to moderately correlated, coinciding with high individual variation in the outcome parameters within each of the methods used to identify the LT2 and RCT. This indicates that an individual with a high LT2 does not necessarily display a high RCT. The high individual variation may be explained by disability-related differences in the cardio-respiratory system that might affect physiological responses to upper-body exercise. For example, athletes with a spinal cord injury exercising in an upper-body mode were shown to vary considerably in their VO_2peak_ depending on their level of injury [44], which might also reflect differences in the % of VO_2peak_ that can be sustained during exercise. In addition, the inclusion of one participant that was much older than the rest and one female participant may have contributed to the high variation. Furthermore, individual variation in physiological responses may be higher in upper-body exercise compared to lower-body exercise. Altogether, it is questionable whether the similar outcome parameters identified at the LT2 and the RCT on a group basis, result in similar outcome parameters at the LT2 and RCT for the individual sitting athlete when training in an upper-body mode.

The thresholds identified by the breakpoints in the VE/VO_2_ at the VT and in VE/VCO_2_ at the RCT did not significantly differ and were highly correlated. This, together with the rather linear increase in the VO_2_-VCO_2_ relationship suggests that it is solely the disproportionate rise in VE that leads to a rather rapid increase in the data of the VE/VO_2_ and the VE/VCO_2_ plots, and to discernible breakpoints in approximately half of the participants. Together with the close location of the breakpoints identified in the VCO_2_-VO_2_ data at the VT and the VCO_2_-VE data at the RCT, this indicates that a two-phase (low-high) rather than a three-phase (low-moderate-high) intensity zone model could be applicable in athletes with a disability who exercise in an upper-body mode. This is in contrast to significant differences between the VT and the RCT in Dekerle et al. [45], who test able-bodied participants in the arm crank ergometry mode, and Leicht et al. [18], who tested wheelchair athletes in the wheelchair treadmill mode. However, our findings are in line with a study of Pires et al. [46], who also found one rather than two thresholds in the gas exchange data in upper-body trained able-bodied participants during exercise in the arm crank ergometry mode. Whether the discrepancies between studies are related to employment of e.g. different populations, protocols or exercise modes needs to be examined further in other experimental designs.

All gas exchange threshold methods have in common that there is an a priori assumption of the presence of a breakpoint, defined as “a place where an interruption or change occurs” [47]. However, the presence or absence of breakpoints in the gas exchange data is a debated topic [8, 12]. Thus, in addition to the breakpoint models used to identify the VT and the RCT in the present study, we fitted continuous no-breakpoint models to the data to investigate if there are clear breakpoints in our data. Here, we found good fit for the breakpoint model used to identify the gas exchange thresholds, but better fit for the curvilinear no-breakpoint models in most cases. We, hence, question if clear breakpoints really exist in the gas exchange data of athletes with disabilities in an upper-body exercise mode.

## Conclusion

In Paralympic athletes who exercise in upper-body poling, the physiological and perceptual outcome parameters identified at the VT and the LT1 showed large differences, which demonstrates that these cannot be used interchangeably to identify the AT. In addition, the close location of the VT, RCT and LT2 does not allow us to distinguish the AT and ANT, indicating that there might only be one threshold in athletes with a disability exercising in an upper-body mode. Furthermore, continuous no-breakpoint models fit the gas exchange data better than breakpoint models in most participants. We, hence, question if clear breakpoints in the gas exchange data really exist in an upper-body exercise mode in athletes with disabilities.

## Supporting information

S1 File Data. Data and analyses conducted in this study of gas exchange and blood lactate threshold in Paralympic sitting athletes.
(XLSX)

S1 Fig VE/VO_2_ and VE/VCO_2_ plots fitted with two regression lines. The data is of the six or seven completed stages of each of the 15 athletes. Breakpoint presence is indicated above each individual plot. Furthermore, it is indicated in the second row above the figures whether the two thresholds occur at the same time, or the VE/VO_2_ occurs before or after the VE/VCO_2_ threshold. *Oxygen uptake (VO*_*2*_*)*, *carbon dioxide production (VCO*_*2*_*)*, *minute ventilation (VE)*.
(TIF)
