# Supplementary figures and images for: Examination of gas exchange and blood lactate thresholds in Paralympic athletes during upper-body poling
Source: PLoS One. 2018 Oct 31;13(10):e0205588. doi: 10.1371/journal.pone.0205588 (PMC6209185; doi:10.1371/journal.pone.0205588)

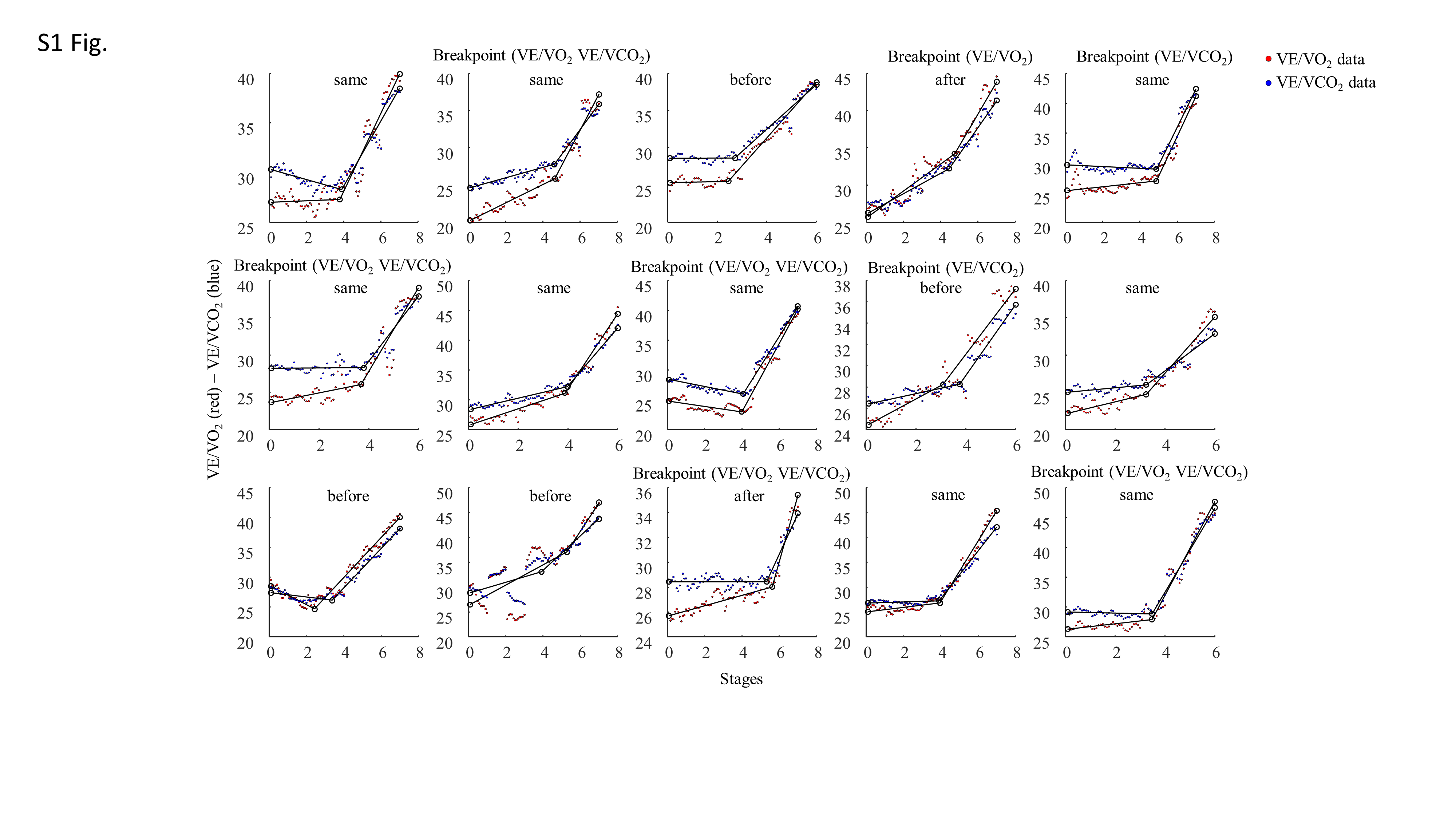

Supplement: S1 Fig — The data is of the six or seven completed stages of each of the 15 athletes. Breakpoint presence is indicated above each individual plot. Furthermore, it is indicated in the second row above the figures whether the two thresholds occur at the same time, or the VE/VO2 occurs before or after the VE/VCO2 threshold. Oxygen uptake (VO2), carbon dioxide production (VCO2), minute ventilation (VE). (TIF) [file pone.0205588.s002.TIF]
